# CODIFI (Concordance in Diabetic Foot Infection): Agreement in reported presence of likely pathogens in swabs and tissue samples from infected diabetic foot ulcers

**DOI:** 10.1186/1757-1146-8-S1-A2

**Published:** 2015-04-20

**Authors:** Michael Backhouse, Andrea Nelson, Alexandra Wright-Hughes, Moninder Bhogal, Sarah Brown, Catherine Reynolds, Benjamin Lipsky, Christopher Dowson, Jane Nixon

**Affiliations:** 1 School of Healthcare, University of Leeds, Leeds, UK; 2 Clinical Trials Research Unit, University of Leeds, Leeds, UK; 3 Division of Medical Sciences, University of Oxford, Seattle, United States; 4 School of Life Sciences, University of Warwick, Coventry, UK

## Aim

Wound infection is common in diabetic foot ulcers with potentially life changing sequelae. Targeted treatment of infective organisms requires accurate identification of pathogens to enable refinement of antibiotic protocols to improve outcomes and reduce antibiotic resistance. Wound sampling is routinely conducted using swabs although some guidelines recommend use of tissue samples. To date there is a lack of robust evidence to inform clinical practice regarding sampling technique.

This study aimed to evaluate the extent to which results from wound swab and tissue samples taken from the same patient agree with each other; one might report pathogens more than the other, they might both report the same pathogens consistently, or they might disagree with a more complex pattern of disagreement. Here we report agreement and disagreement between the techniques based upon reported presence of likely pathogens.

## Methods

In this multi-centre, cross-sectional study, 401 patients with a suspected infected diabetic foot ulcer were recruited from 25 sites across England. All patients had both a swab and tissue sample taken from same ulcer for plating and culture. Agreement between techniques in the reported presence of likely pathogens was assessed by overall prevalence and bias adjusted Kappa (PABAK). McNemar’s test was used to investigate patterns of disagreement.

## Results

401 patients were recruited between 2011 and 2013 with a median age of 63 years; 79% were male; 85.5% had type 2 diabetes; 27.5% presented with a recurrent ulcer; and 45.5% had a neuro-ischaemic ulcer, 50.5% neuropathic, and 3.5% ischaemic.

Both swab and tissue reports were available for 395 patients, and at least one pathogen was reported in 70.1% of swab samples and 86.1% of tissue samples. In 58% of patients the two samples resulted in a difference in the reported pathogens, with: 13.2% both reporting different pathogens; 36.7% reporting additional pathogens in the tissue sample; and 8.1% reporting additional pathogens in the swab sample.

In the most prevalent pathogens (those present in >8%), there were significantly higher rates of reporting of the following pathogens from tissue samples than swabs (McNemar’s p-value <0.05): Gram Positive Cocci, Gram Negative Bacilli, Enterobacteriacea, Anaerobes, Streptococcus, Enterococcus, Coagulase-Negative Staphylococcus, Gram Positive Bacilli, Corynebacterium; with differences ranging between 3.3% (Streptococcus) and 13.7% (Gram Positive Cocci). There was no evidence of a difference in reporting for Methicillin-resistant S. Aureus (MRSA) (p=0.22); Staphylococcus Aureus and Pseudomonas (the latter two both had p=1.00). In terms of agreement, the PABAK ranged from 0.58 (Gram Positive Cocci) to 0.97 (MRSA).

Investigation of the influence of baseline factors on agreement of the number of reported pathogens found, despite large centre variation, significant evidence (p=0.02) of an association with ulcer duration, such that older ulcers ( ≥56 days old) had a reduced odds (0.64 95% CI (0.45, 0.95)) of the tissue sample reporting more pathogens than the swab.

## Conclusions

Overall, there were significant differences in the pathogens reported from swab and tissue samples in patients with suspected infected diabetic foot ulcers. This has potential implications for choice of sampling technique in clinical practice.

